# Synthesis of functionalized fluorescent silver nanoparticles and their toxicological effect in aquatic environments (Goldfish) and HEPG2 cells

**DOI:** 10.3389/fchem.2013.00029

**Published:** 2013-12-05

**Authors:** Elisabete Oliveira, Hugo M. Santos, Javier Garcia-Pardo, Mário Diniz, Julia Lorenzo, Benito Rodríguez-González, José L. Capelo, Carlos Lodeiro

**Affiliations:** ^1^Bioscope Group, REQUIMTE, Chemistry Department, Faculty of Science and Technology, University Nova of LisbonLisbon, Portugal; ^2^Veterinary Science Departments, CECAV, University of Trás-os-Montes and Alto DouroVila Real, Portugal; ^3^Institut de Biotecnologia i Biomedicina and Departament de Bioquímica i de Biologia Molecular, Universitat Autònoma de BarcelonaBellaterra, Barcelona, Spain; ^4^Scientific and Technological Research Assistance Centre (CACTI), University of VigoVigo, Spain

**Keywords:** silver nanoparticles, fluorescence, cytotoxicity, goldfish, human hepatoma cells

## Abstract

Silver nanoparticles, AgNPs, are widely used in our daily life, mostly due to their antibacterial, antiviral, and antifungal properties. However, their potential toxicity remains unclear. In order to unravel this issue, emissive AgNPs were first synthetized using an inexpensive photochemical method, and then their permeation was assessed *in vivo* in goldfish and *in vitro* in human hepatoma cells (HepG2). In addition, the oxidative stress caused by AgNPs was assessed in enzymes such as glutathione-S-transferase (GST), catalase (CAT), and in lipid peroxidation (LPO). This study demonstrates that the smallest sized AgNPs@**3** promote the largest changes in gold fish livers, whereas AgNPs@**1** were found to be toxic in HEPG2 cells depending on both the size and functionalized/stabilizer ligand.

## Introduction

Silver nanoparticles (AgNPs) have gained momentum due to their use in imaging, catalysis, electronics, photonics, and photography (Evanoff and Chumanov, 2005; Dahl et al., 2007; Liang et al., 2007; Pastoriza-Santos and Liz-Marzán, 2008). In addition, medical devices and therapeutic products containing AgNPs also offer health benefits, such as antibacterial, antiviral, and antifungal properties. Because of such properties, AgNPs are of the focus of precautions and regulations related to public health and environmental risks (Faunce and Watal, 2010). In the USA there are more than 260 commercialized products containing AgNPs, such as cleaners, clothing, and personal care products (Faunce and Watal, 2010). Silver itself is known to be highly toxic in cells, but AgNP toxicity still has been target of studies. AgNPs can form protein-silver complexes, which according to *in vitro* studies, can be deposited in the liver, kidney, lungs, brain, and/or skin, as they are highly toxic to mammalian liver cells, stem cells, and brain cells (Hussain et al., 2005; Landsdown, 2007).

The US National Institute for Occupational Safety and Healthy (NIOSH) has established an exposure limit of 0.01 mg/m^3^ for all forms of silver (Drake and Hazelwood, 2005). In 2010, nanosilver was considered one of the 15 issues that can negatively affect the conservation of biological diversity (Sutherlandm et al., 2010). The experts are concern about the wide use of nanosilver, not mainly because of the direct poisoning in humans or production of bacterial resistance in hospital setting, but due to their highly *in vitro* toxicity for aquatic organisms and capability to persist in the environment. Silver and its forms can be release in the environment into waste streams, such as from photographic development, which has been the major source of ecological toxicity (Luoma, 2008).

Toxicological studies of Ag nanoparticles with different diameters, 36, 52, and 66 nm have been reported in freshwater environments through the use of *Daphnia magna*. It was found that within the concentration range of 3–4 μ g/L, toxicity is not size-dependent (Li et al., 2010). Comparative studies of gold and AgNPs reveal that the silver ones are the most toxic (Hussain et al., 2005). Regarding AgNP synthesis, the most common and straightforward approach is based on reductive methods, using sodium borohydrate, sodium citrate, or irradiation (Lee and Meisel, 1982; Chau et al., 2005). To avoid particle aggregation, this is often done in the presence of additives such as organic molecules, peptides (Lodeiro et al., 2010; Oliveira et al., 2011a,b), or polymers. Usually metal nanoparticles do not present fluorescence emission, due to the electron transfer from the AgNP metal core to the additive.

On the other hand in some cases, the presence of other types of additives in the surrounding AgNPs enhances their photophysical properties, such as luminescence (Tam et al., 2007). Research into fluorescent nanoparticles has thus, increased steadily in the last few years, because fluorescence can be traced *in vivo* and *in vitro* (Alivisatos, 1996). AgNPs exhibit an intensive surface plasmon resonance (SPR) band, due to the collective oscillation of the electrons, in the wavelength range of 300–900 nm (Díez et al., 2009). The localization of the SPR band depends on several parameters, such as, size, shape and the refractive index of the environment (Si et al., 2007; Belser et al., 2009).

Maretti et al. (Maretti et al., 2009) published a new easy method based on the photochemical synthesis of fluorescent AgNPs, which can be used in imaging applications. In Maretti's method, the AgNPs were stabilized by cyclohexylamine, and the metal is reduced by ketyl radicals.

For “Proof of Concept” of the study viability in a complex organism and in cell cultures, AgNP permeation and toxicity were carried out *in vivo* in gold fish (*Carassius auratus*), whilst cytotoxicological studies were performed *in vitro* in human hepatoma cells. AgNPs were synthetized with a new easy and inexpensive method, which was developed by varying Maretti's method (Maretti et al., 2009), using **(1)** 5-Aminoisoquinoline, **(2)** 7-amino-4-methylcoumarin, and **(3)** 2-Aminoanthracene as additives. AgNP formation was monitored by UV-Vis and fluorescence emission spectroscopy, matrix-assisted laser desorption ionization time of flight mass spectrometry (MALDI-TOF MS), dynamic light scattering (DLS), transmission electron microscopy (TEM), and high-resolution transmission electron microscopy (HRTEM).

## Materials and methods

### Chemicals and starting materials

5-Aminoisoquinoline, 7-amino-4-methylcoumarin, and 2-Aminoanthracene, 2-Hydroxy-4′-(2-hydroxyethoxy)-2-methyl-propiophenone (I-2959) were commercially from Aldrich. Ag(CF_3_SO_3_) was from Fluka. All were used without further purification.

### Physical measurements

#### Instruments

The samples were exposed to ultraviolet light at 365 nm, in a common UVA lamp, 230 V, 50 Hz, Serial n° MO31975, Ambientáls.

#### Particles size distribution

The nanoparticle size distributions were measured using DLS, a Malvern Nano ZS instrument with a 633 nm laser diode.

#### TEM measurements

To perform the TEM images, the samples were prepared by dropping 1 μL of the colloidal suspension onto a copper grid coated with a continuous carbon film and allowing the solvent to evaporate. TEM and HRTEM images were obtained using a JEOL JEM 2010F transmission electron microscope (TEM) operating at 200 kV. To perform the Fourier transformations, we used Digital Micrograph (Gatan) software. Data for size distribution histograms were obtained by measuring more than 100 particles per sample in several TEM images (Saìnchez-Iglesias et al., 2006).

#### MALDI-TOF-MS studies

The MALDI-MS analyses were performed in a MALDI-TOF-TOF-MS model Ultraflex II Bruker, Germany, equipped with nitrogen. Each spectrum represents accumulations of 5 × 50 laser shots. The reflection mode was used. The ion source and flight tube pressure were less than 1.80 × 10^−7^ and 5.60 × 10^−8^ Torr, respectively. The MALDI mass spectra of the soluble samples (1 or 2 μ g/μ L) were recorded using the conventional sample preparation method for MALDI-MS. 1 μ L of ligand was placed on the sample holder. The sample holder was inserted in the ion source.

#### Photophysical measurements

Absorption spectra were recorded on a JASCO V-650 spectrophotometer and fluorescence emission on a FLUOROMAX-4 HORIBA SCIENTIFIC spectrofluorometer.

#### Synthesis of silver nanoparticles

In an quartz cell 1.02 × 10^−2^ M of I-2959, 3.4 × 10^−3^ M of Ag(CF_3_SO_3_), and 2.0 × 10^−4^ M of compounds **1**, **2**, or **3**, were added to a 3 mL of THF solution. The spectral changes were observed upon irradiation. Thereafter, the nanoparticles obtained were washed several times by centrifugation, in order to remove the unreacted products.

#### Cell culture

Human hepatocellular carcinoma HepG2 cell line (ATCC HB-8065), derived from human hepatocytes, was maintained in a Minimum Essential Medium (MEM) alpha medium supplemented with 10% (v/v) heat inactivated fetal bovine serum (FBS) in a highly humidified atmosphere of 95% air with 5% C0_2_ at 37°C.

#### Cytotoxicity of the AgNPs

*The cytotoxicity of* AgNPs (**1**, **2**, and **3**) was tested in human HepG2 cells. Cells were seeded into a 96-well plate at a cell density of 6.0 × 10^3^ cell/well and incubated for 24 h before AgNPs were added at a concentration of 25 and 100 μg/ml. The growth inhibitory effect was measured after 72 h treatment by the XTT assay (Cory et al., 1991; You et al., 2012). Aliquots of 20 μ l of XTT solution [2,3-bis-(2-methoxy-4-nitro-5-sulfophenyl)-2H-tetrazolium-5-carboxanilide] were added to each well. After 3 h, the color formed was quantified by a spectophotometric plate reader (Perkin Elmer Victor^3^ V) at a 490 nm wavelength. Cell cytotoxicity was evaluated in terms of cell-growth inhibition in treated cultures and expressed as a % of the control conditions. Trypan Blue is one of the many dye recommended exclusion staining, counting and evaluation of cellular population and acute cellular toxicity (Wong et al., 2006) techniques. This method is based on the principle that the living cells do not incorporate the dye, whereas dead cells incorporate it owing to their damaged membrane. Cells treated with 100 μg/ml concentration of AgNPs for 72 h were rinsed with PBS, stained with 0.2% Trypan Blue (Invitrogen) for 5 min and observed by microscopy.

#### CLSM in vivo imaging of AgNP-treated HepG2 cells

HepG2 cells were seeded at a density of about 2 × 10^5^ cells per plate on 14 mm glass bottom microwell dishes (MatTek, corp). After 24 h cells were treated with 25 and 100 μg/ml of AgNPs (**1**, **2**, and **3**) and incubated for 24 h. At the end of incubation, cells were washed three times with media and stained for 7 min with Hoechst (Invitrogen) and CellMask red (Invitrogen) at 37°C. Imaging was performed immediately at 37°C and 5% CO_2_ on a Leica TCS SP5 confocal microscope using a 63 × 1.4 numerical aperture Plan Apochromat oil-immersion lens. Acquired images were processed using Bitplane Imaris 7.2.1 software.

#### Flow cytometry of AgNP-treated cells

The cellular uptake of AgNPs (**1**, **2**, and **3**) was carried out via flow cytometric measurements. To perform the experiments 3.0 × 10^5^ HepG2 cells per plate were seeded in 6-well plates (BD Falcon). After 24 h treatments with 25 and 100 μg/ml of AgNPs (**1**, **2**, and **3**), cells were collected and centrifuged. Cells were then rinsed and resuspended in 1 ml of PBS. Flow cytometry analysis was performed with FACSCalibur flow cytometry at an emission wavelength of 488 nm.

#### In vivo studies

Goldfish (*Carassius auratus*) were obtained from local producers (*N* = 6 per treatment, average weight: 10.2 ± 3.4 g) and acclimated for 2 weeks to laboratory conditions in 400 L polystyrene. The fish were housed in a closed circuit system with filtered tap water at a temperature of 20 ± 1°C, pH 7.2 ± 0.2, and continuous aeration and fed daily with commercial feed flakes (TetraMin). Following synthesis, AgNps were injected in fish by an intraperitoneal injection to evaluate their ability of internalization in fish tissues. Thus, two assays (**A** and **B**) with a total of 24 animals were carried out. **A**—Intraperitoneal injection of AgNPs in PBS (1:10); **B**—Intraperitoneal injection of AgNPs diluted in corn oil (1:10). After injection, fish were maintained in 15 L polystyrene tanks, with de-chlorinated tap water, photoperiod 12:12 h light/dark (L/D and continuous aeration (dissolved O_2_ > 6 mg/L). Control fish were housed in the same laboratory conditions but were injected intraperitoneally with (**a**) a solution of PBS (phosphate buffer solution), (**b**) a solution of PBS and THF (1:10); and (**c**) a solution of PBS and corn oil (1:10). After 48 h fish, including controls, were sacrificed by cervical sectioning and dissected with the aid of scissors, a scalpel and forceps, and the liver and intestine removed. Samples from organs were stored at −80°C for further analysis and other subsamples were taken for histological processing.

Total Glutathione-S-Transferase (GST) activity was determined as described by Habig et al. (Habig et al., 1974) by measuring the formation of the conjugate of glutathione (GSH) and 1-chloro-2,4-dinitrobenzene (CDNB). Briefly, 180 μ L of substrate solution (Dulbecco's Phosphate Buffered Saline with 200 mM L-glutathione reduced and 100 mM CDNB all from Sigma-Aldrich, Germany) were added to 20 μ L of GST standard or sample into each well of a 96-well microplate. The total enzyme activity was determined at 340 nm by recording the absorbance at every minute for 6 min, using a microplate reader (BioRad Benchmark, USA). Equine liver GST (Sigma-Aldrich, Germany) was used as standard and positive control. The change in absorbance per minute (λ A_340_) was estimated and the reaction rate at 340 nm was determined using CDNB extinction coefficient of 0.0096 μ M^−1^cm^−1^. The results are expressed in relation to total protein concentration of the sample (nmol min^1^mg^1^ total protein).

Catalase(CAT) activity was determined as previously described by Aebi (Aebi, 1984), which follows the decrease in absorbance at 240 nm by H_2_O_2_ consumption. Briefly, A substrate solution of 0.036% (w/w) H_2_O_2_ was prepared in buffer 50 mM KH_2_PO_4_ (Sigma-Aldrich, Germany) pH 7.0 containing 1 mM EDTA (Riedel-Haën, Germany), at 25°C using 30% (w/w) H_2_O_2_ (Sigma-Aldrich, Germany). To perform the assay, 0.1 mL of CAT standard or sample were added to 2.9 mL of the substrate solution in individual quartz cuvettes and absorbance at 240 nm was recorded every 30 s for 180 s (at 25°C, pH 7.0, and path length 10 mm), using a spectrophotometer (Unicam Helios, UK). The consumption of peroxide was monitored using a extinction coeff. 0.04 mmol^−1^cm^−1^. Bovine liver CAT (Sigma-Aldrich, Germany) was used as standard and positive control.

Lipid peroxides assay adapted from TBARS (thiobarbituric acid substances) protocol was determined by the quantification of a specific end-product of the oxidative degradation process of lipids, the malondialdehyde (MDA). In this assay thiobarbituric acid reacts with MDA to produce a fluorescent product detected spectrophotometrically at 532 nm. Briefly, samples were treated with 8.1% dodecyl sulfate sodium, 20% trichloroacetic acid (pH 3.5), thiobarbituric acid, mixture of n-butanol and pyridine (15: 1, v/v) (Correia et al., 2003). To quantify the lipid peroxides, MDA concentrations were calculated with the computer program Microplate Manager 4.0 (BIO-RAD, USA) based on an eight-point calibration curve (0–0.3 μM TBARS) using MDA bis(dimethylacetal) (from Merck). The enzymatic results are expressed in relation to total protein of the sample (nmol/min/mg) calculated following the procedure described by Bradford (Bradford, 1976) using albumin as a standard.

The histological procedures were carried out essentially according to Martoja and Martoja (Martoja and Martoja, 1967). Briefely, after being fixed in Bouin-Hollande's for 48 h, samples were washed in distilled water and dehydrated through a series of graded ethanol solutions and toluene (Lab-Scan, Belgium) for intermediate impregnation. Organs were embedded in paraffin (Panreac, Spain), cut in sections of 5 μm thickness and mounted in glass slides. Paraffin was removed from slides using xylene as solvent, followed by rehydration in a graduate series of alcohols, washing with demineralized water, then glycine (0.3 M) and staining (5 min.) with toluidine blue-O (0.5%) prepared from a solution of NaBH_4_ (1%) for further histological analysis. Staining with toluidine blue O allows quenching of auto-fluorescence and enhances the fluorescence of nano-emissive particles (Kiernan, 2008). The presence of emissive NPs within tissues was assessed using a Leica microscope (Leica-ATC 2000, Germany), with an image system from Leica Microsystems (DMLB model) adapted for epifluorescence and equipped with an EL6000 light source for mercury short-arc reflector lamps was used. For tissue microscope analysis I3 (blue) and N2.1 (green) filters were employed.

#### Statistical analysis

The results were expressed as the mean ± SD. The statistics of enzymes activities were performed by the non-parametric test Kruskal-Wallis, with a significant level of *p* < 0.05, using the software *Statistica* 8.0 (StatSoft Inc., USA).

## Results and discussion

**Figure 1:**
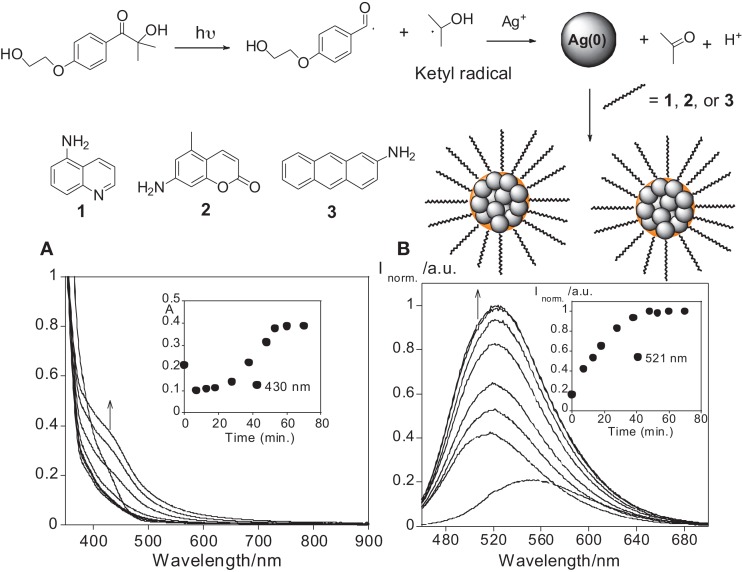
**Above:** General synthetic pathway of the AgNPs synthesis. **Below: (A)** Absorption and emission **(B)** spectra of the AgNPs formation with time containing as additive compound **1**, in a THF solution, *T* = 298 K, λ_exc_ = 430 nm.

The photosynthesis of the AgNPs was performed in a quartz cell followed by the addition of I-2959, AgCF_3_SO_3_, and the chromophore (**1**, **2**, or **3**) in a THF solution, with subsequent exposure to a UVA lamp at 365 nm. The I-2959 acts as a source of ketyl radicals, which are known to be strong reducing agents, allowing the reduction of silver ions in the presence of **(1)** 5-Aminoisoquinoline, (**2**) 7-amino-4-methylcoumarin, and **(3)** 2-Aminoanthracene (see Figure 1). The chosen compounds **1–3** have all their absorption spectrum in the UV region, since the lamp used for photochemical synthesis *via* irradiation was at λ = 365 nm. Moreover, all compounds present an amine group in the chemical skeleton in order to be used for surface nanoparticle attachment.

After UV irradiation at λ = 365 nm, the AgNPs with compound **1** showed a red shift from 410 to 430 nm, and an increase in absorbance at 430 nm up to 40 min after UV exposition (see Figure 1A). Similar behavior over time has been reported in previous studies on the nucleation of gold nanoparticles; in these works the abrupt jump in absorbance corresponds with the particle nucleation burst (Chow and Zukoski, 1994; Rodríguez-González et al., 2007). In the emission spectra, a blue shift from 550 to 521 nm was observed, as well as an enhancement in the emission intensity at 521 nm (see Figure 1B). The growth plot also shows a long tail in the range 550–600 nm after 30 min (see Figure 1A). This is most likely due to the formation of larger AgNPs aggregates. Interestingly, the maximum emission intensity observed was reached at 40 min. This matches the nucleation burst time as highlighted by the plot of absorbance as a function of time (see Figure 1A).

Nanoparticles stabilized with compound **2**, AgNPS@**2**, showed an increase in the absorbance detected at 420 nm up to 20 min after irradiation. However, at values up to 10 min, a new small band at 600 nm was observed, also revealing the presence of nanoparticle aggregates (see Figure SI1A). Once again, there was an emissive band at 510 nm (see Figure SI1B).

A similar behavior was obtained for AgNPs@**3**, with the appearance and increase in the absorbance at 450 nm and the emission at 490 nm. However, an exposure of longer than 10 min UV light induced nanoparticle aggregation, as only 10 min was the best time for nanoparticle formation. Overall nanoparticle formation was confirmed in this case by the greenish emission, and was also detected be the naked eye by the change from *colorless* to *yellow.* The yellow color of the dispersion is a strong indication of nanoparticle formation, because it is related to the appearance of the plasmon band in the UV region, which is characteristic of AgNPs. TEM images revealed that the AgNPs obtained were almost spherical.

**Figure 2:**
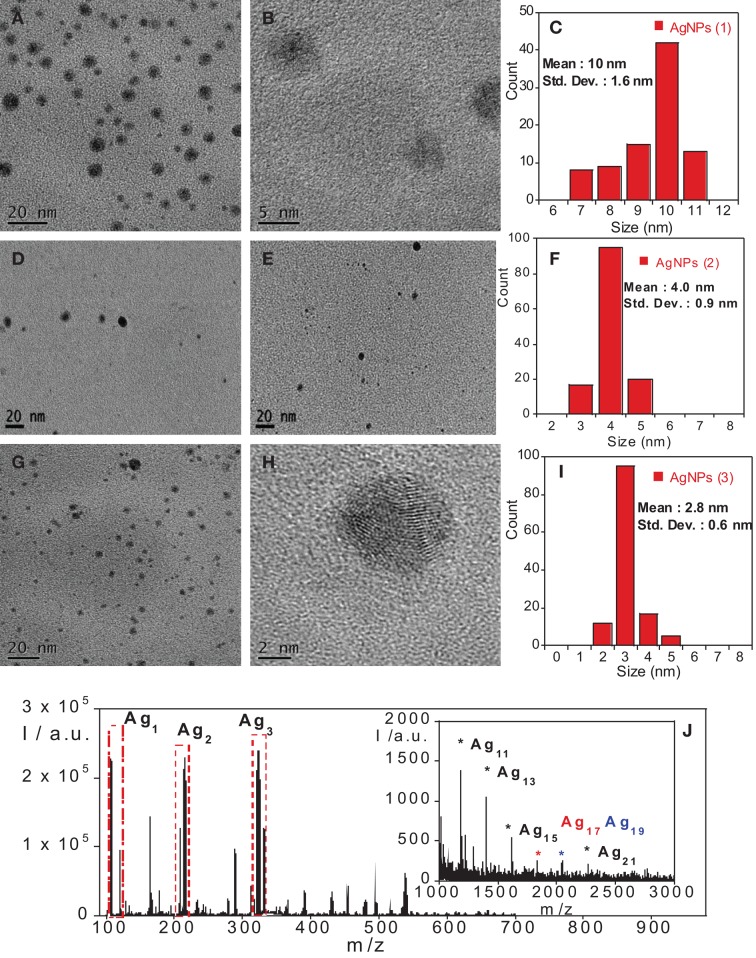
**(A,D,E,G)** TEM image of AgNPs with compound **1 (A), 2 (D,E)**, and **3 (G). (B,H)** High-resolution transmition electron microscopy (HRTEM) image of AgNPs with **1 (B)** and **3 (H)**. Size histogram of nanoparticles with **1 (C), 2 (F)**, and **3 (I). (J)** MALDI-TOF MS spectra of AgNPs with compound **1** in THF without matrix.

Average sizes of 10 ± 1.6, 4.0 ± 0.9, and 2.8 ± 0.6 nm, were obtained from TEM images for the AgNPs with additives **1**, **2**, and **3**, respectively, (see Figure 2).

AgNP stability was confirmed by the zeta potential, where the values of −40 ± 5 mV, −38 ± 4 Mv, and −50 mV ± 5 mV indicated high stability, for **1**, **2**, and **3**, respectively. Taking into account the spectral data mentioned above, it was concluded that the additive directly influences nanoparticle formation. This is most likely due to the increasing number of donor atoms, which leads to a displacement in the emission wavelength, as well as an increase in nanoparticle size.

Figures 2A–I shows the TEM, HRTEM and size histogram of the AgNPs synthetized in this work. The histograms were obtained from multiple TEM images such as those shown in Figure 2. The histograms show a small difference in size distribution, which is due to the different influence of **1** to **3** additives on the silver nanoparticle fabrication.

The particles shown in Figure 2 are larger than the clusters reported in Maretti's work (Maretti et al., 2009). According to the literature (Liz-Marzán, 2006; Pompa et al., 2006) the collective oscillation of the electrons leads to the appearance of an absorption band, known as a plasmon band. It is well known that the plasmon absorption band does not involve the presence of an emission band, which is why in bare nanometer sized silver particles are not found in any emission band.

The question that now arises is what is the origin of the observed fluorescence in these samples? Some authors have pointed to the presence of silver clusters (Ag_*n*_, *n* = 2–8) in the samples as the main source of the emission band (Zhang et al., 2005; Vosch et al., 2007). Maretti reports that the clusters are predominantly of type Ag_2_ and are likely to be supported by larger nanometer sized particles, such as the particles shown in Figure 2. In our case highly emissive AgNPs were also obtained, thus, the emission could be attributed to the formation of small clusters, such as, Ag_2_, Ag_3_, and Ag_4_ attached to the nanometer sized nanoparticles shown in Figure 2. The joining of nanoparticles and clusters on the surface could form a very strong emission ensemble (Maretti et al., 2009; Rao and Pradeep, 2010). Due to the small size of the clusters and their relatively low stability under the electron bean, it is quite difficult to image them by HRTEM.

### MALDI-TOF-MS studies

In order to confirm the clusters hypothesis, we carried out mass spectrometry experiments to demonstrate the presence of Ag clusters in the samples. In all cases cluster formation was confirmed by MALDI-TOF MS. Peaks at 108.0, 215.2, 322.3, 970.0, 1186.0, 1401.9, 1617.7, 1833.5, 2049.3, 2265.2 m/z, corresponding to the species Ag_1_, Ag_2_, Ag_3_, Ag_9_, Ag_11_, Ag_13_, Ag_15_, Ag_17_, Ag_19_, and Ag_21_ for compound **1** (see Figure 2J) were found. The results also revealed that stabilizers do not affect cluster formation, where the same silver species were found for all the compounds.

#### Cytotoxicological studies in goldfish

Depending on their size, AgNPs could permeate through skin and the intestine, and then be accumulated in the liver, lung, blood, kidney, and/or stomach. In order to evaluate the permeation and toxicity of nanoparticles, cytotoxicological studies were performed *in vitro* in human hepatoma cells and *in vivo* studies in goldfish (*Carassius auratus*). The studies *in vivo* were carried out by analyzing oxidative stress [lipid peroxidation (LPO) and CAT], phase II biotransformation of xenobiotics GST, and histological examination.

**Figure 3:**
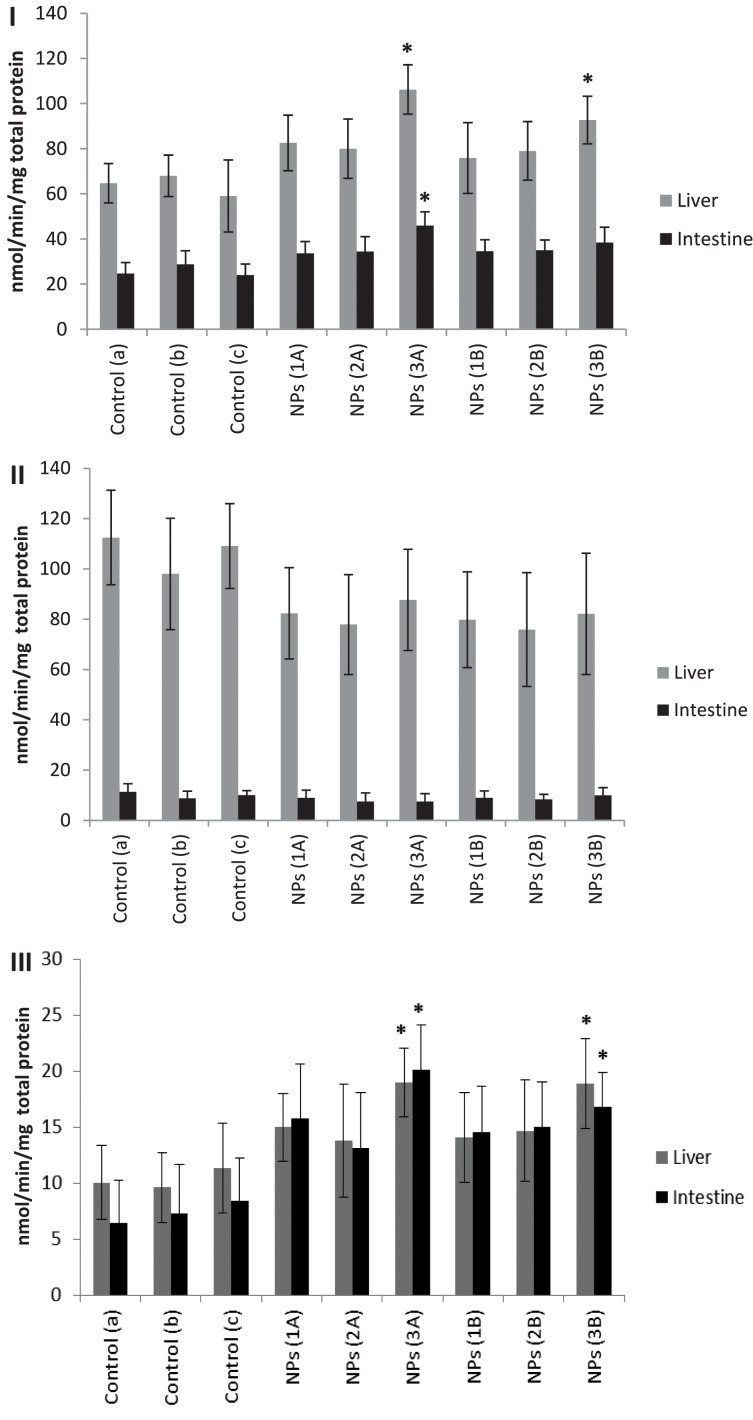
**(I) GST, (II) CAT, and (III) MDA concentrations in the liver and intestine tissues of *Carassius auratus* treated with AgNPs for 48 h**. Data are presented as means ± SD. The asterisks indicate significant differences (*p* < 0.05) in comparison to controls. A—Intraperitoneal injection of AgNPs in PBS (1:10); B—Intraperitoneal injection of AgNPs diluted in corn oil (1:10). Legend: control (a) PBS (phosphate buffer solution), control (b) a solution of PBS and THF (1:10); and control (c) a solution of PBS and corn oil (1:10).

Enzymes such as GST or CAT are part of the antioxidant system responsible for the elimination of radicals and molecules produced as a consequence of oxidative stress. The GST is a family of phase II detoxification enzymes that catalyze the conjugation of glutathione (GSH) to a wide variety of endogenous and exogenous electrophilic substances, such as drugs, toxins, and products of oxidative stress, and therefore, they play an important role in preventing oxidative damage (Habig et al., 1974; Hayes et al., 2005). The results of the GST activity measured in the intestines and liver of fish treated with the AgNPs are shown in Figure 3. The GST levels determined in livers were higher than in the intestines. This is because liver is the major metabolic and detoxification organ in living organisms.

The results show a general trend to increase GST activity in injected fish compared to controls. Statistical analysis of the results of GST activity in fish organs (Figure 3I) revealed significant differences (*p* < 0.05) between controls, intestines and livers from fish injected with the AgNPs **3**A (diluted in PBS). For fish injected with AgNP **3**B (diluted in corn oil) significant differences were found in livers compared to controls. These results suggest that fish injected with AgNPs **3** are subjected to higher oxidative stress than when they are injected with other AgNPs.

In a different study, Chae et al. (Chae et al., 2009) tested two different AgNP dosages in Japanese medaka (*Oryzias latipes*) and quantified GST (as well as other markers) by measuring the mRNA concentrations in liver extracts. They found that these two AgNPs led to high toxicity thus, causing cellular and DNA damage, as well as carcinogenic and oxidative stresses. In contrast, Kwok et al. (Kwok et al., 2012) showed that different AgNP coatings (citrate, gum Arabic and polyvinylpyrrolidone) influence toxicity in the early life of Japanese medaka. Exposure assays showed that citrate and polyvinylpyrrolidone exhibited similar and lower toxicity, however, all the AgNPs coated were three to ten times less toxic than AgNO_3_.

The decomposition of H_2_O_2_ (Maehly and Chance, 1954) produced in living organisms as a consequence of oxidative stress takes place in a chemical reaction that involves CAT. CAT acts as the catalyst of the reaction. The presence of Ag (I) ions has been shown to lead to a deformation in Zebrafish embryos, as well as increase in CAT concentration (Choi et al., 2010). In addition, Ag (I) ions are 300 times more toxic to Zebrafish than AgNPs (Scown et al., 2010; Powers et al., 2011). On the other hand, no significant CAT response in the hepatocyte cultures of fish exposed to AgNPs was observed (Scown et al., 2010). In our studies no differences were found between injected fish and controls, as shown in Figure 3II.

LPO has been shown to cause various negative effects in terms of cellular integrity in the membranes of the cells (which may lose permeability and function) as well as other changes such as the production of pro-inflammatory agents and potentially toxic substances (Greenberg et al., 2008). LPO is commonly used as an indicator of oxidative stress in cells and tissues (Botsoglou et al., 1994). An increase in LPO in the intestine and livers was observed for the fish injected, as shown in Figure 3III. However, significant differences (*p* < 0.05) were detected only in intestines and livers from fish injected with AgNPs **3**A and **3**B compared to controls. These results are generally in agreement with GST and CAT results.

Livers from all the controls showed normal histology, while livers from fish injected with AgNPs presented morphological changes varying from subtle to moderate according to the type of AgNP (e.g., tissue degeneration, chromatin condensation, pyknosis) since AgNP **3** cause larger significant changes in livers. No significant changes in tissues were detected by the histological examination of the intestines. However, other authors have found alterations in tissues or cells following exposure to AgNPs (Choi et al., 2010; Farmen et al., 2012).

In order to verify the toxicity of stabilizers **1** to **3**, analysis of oxidative stress measured by GST and CAT activities and LPO (MDA content) as indicator of cell damage were carried out. The intraperitoneal injection of 0,0075 mg/g fish weight of stabilizers **1**–**3**, diluted in PBS (1:10), from the statistical point of view do not reveal a significant increase in antioxidant enzymes activities for both organs (liver and intestine). However, a trend for antioxidant enzymes and MDA increase was observed in livers from fish injected with stabilizer 3. Thus, the results suggest that the concentrations of compounds injected present no significant toxicity to organisms (see Figure SI2).

**Figure 4:**
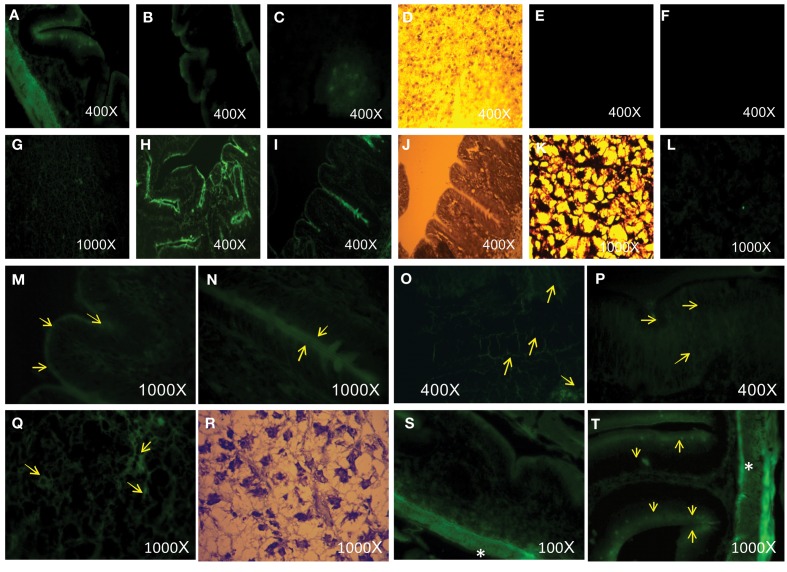
**(A,B)** AgNPs with compound **1** in fish intestine: green fluorescence. THF: In fish intestine **(E)** and liver **(F)**: no fluorescence visible. **(C)** AgNPs with compound **1** in fish liver: green fluorescence. **(D)** Bright field optical microscopy of liver (same spot of **C**). **(G,L)** AgNPs with compound **2** in fish liver: green fluorescence. **(I,H)** AgNPs with compounds **2** in fish intestine: green fluorescence. **(K,J)** Bright field optical microscopy of liver **(K)** and intestine **(J)** (same spot of **G** and **I**, respectively.). **(M–P,S,T)** AgNPs with compound **3** in fish liver: green fluorescence (arrows). **(Q,R)** AgNPs with **3** in fish liver: green fluorescence (arrowhead as an example). **(R)** Bright field optical microscopy of liver (same slide spot of **Q**). (^*^) The more intense fluorescence at the external muscular layers.

Histological observation by epi-fluorescence microscopy showed that there is evidence of nanoparticles in intestine and liver cells, which was revealed by the presence of small fluorescent clusters (Figure 4) suggesting that the AgNPs had penetrated the tissues and cells.

#### Dose-dependent cytotoxicity of AgNPs

**Figure 5:**
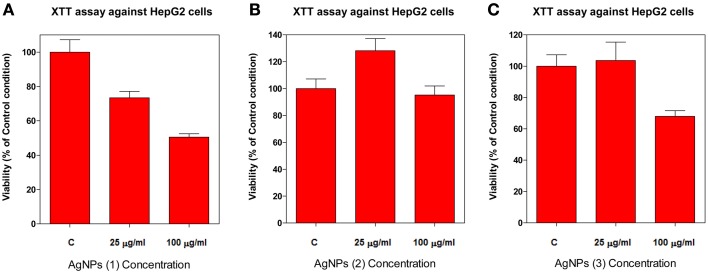
**Dose-dependent cytotoxicity of AgNPs against HepG2 cells**. The cytotoxic effect of AgNPs against HepG2 cell line was evaluated by the XTT assay. Cell viability was evaluated after 72 h treatment with two different nanoparticle concentrations (25 and 100 μg/ml): compound **1 (A)**, compound **2 (B)** and compound **3 (C)**.

The viability of HepG2 cells, after exposure to AgNPs stabilized with compounds **1**, **2**, and **3**, was examined by XTT (Cell Proliferation Assay Kit) assays. The assay measures the amount of XTT reduction by mitochondrial dehydrogenase and assumes that cell viability (corresponding to reductive activity) is proportional to the production of purple formazan which is measured spectrophotometrically. Exponentially divided HepG2 cells were treated with two concentrations (25 and 100 μg/ml) of each AgNP for 72 h, as described in the experimental section. The result from the XTT assay showed that AgNPs differ greatly in their cytotoxic properties depending on their stabilizer compound in a dose-dependent manner (Figure 5).

We found that AgNPs stabilized with compound **1** exerted a cytotoxic effect on HepG2 cells. As shown in Figure 5A, the viability of HepG2 cells exposed to these particles for 72 h, decreases from 73.4% at 25 μg/ml, to 50.5% at 100 μg/ml, when compared with the control (*p* < 0.05). AgNPs stabilized with compound **3** displayed low toxicity at doses up to 100 μg/ml, showing a decrease to 68.0% in cell viability (Figure 5C). On the other hand, no cytotoxic effect was observed with AgNPs stabilized with compound **2** when up to 100 μg/ml concentration was assayed (Figure 5B).

Since certain nanoparticles have been suggested to interfere with the XTT assay, HepG2 cells were treated with AgNPs stabilized with compounds **1**, **2**, and **3** at 100 μg/ml and studied under an optical microscope after staining with trypan blue. Captured images of treated cells with AgNPs stabilized with compound **1** showed a high number of non-viable cells (see Figure SI3C). Optical images of HepG2 cell treatments with AgNPs stabilized with compounds **2** and **3** did not show significant changes when compared with control cells (Figure SI3C and Figure SI3D), in accordance with the results obtained with the XTT assay (Figure SI3E).

#### Intracellular location of AgNPs

**Figure 6:**
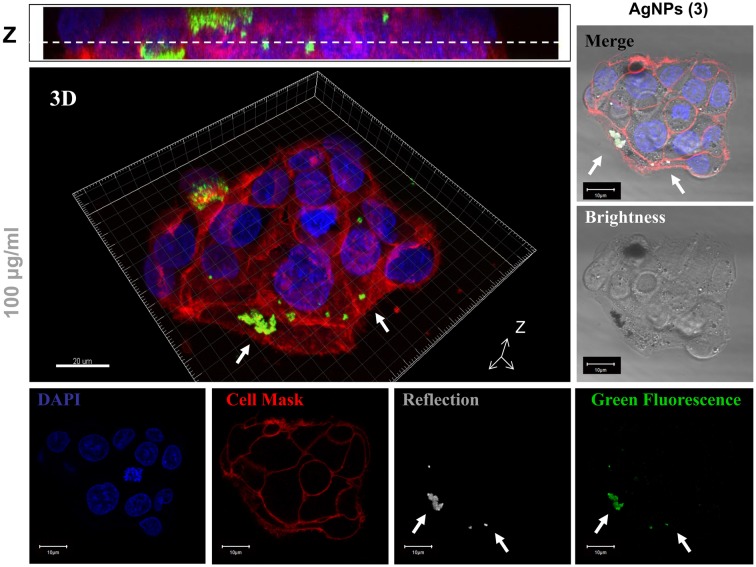
**Confocal Laser Scanning Microscopy (CLSM) *in vivo* images of internalized AgNPs (stabilized with compound 3) in HepG2 cells after 24 h of treatment at 100 μg/ml**. Confocal images obtained of a single channels of nanoparticle fluorescence (green fluorescence), nanoparticle reflection brightness (gray), nuclear stain (DAPI), and membrane stain (Cell Mask) are shown. Merge images of all individual channels (merge), three dimensional reconstruction (3D) and Z projection (Z) allows to visualize the AgNPs particles into HepG2 treated cells. White arrows indicate the location of internalized AgNPs.

To investigate the biodistribution as well as the ability of AgNPs to enter cells, we examined HepG2-treated cells with 100 μg/ml of each AgNP for 24 h using confocal laser scanning microscopy (CLSM). Using CLSM, AgNPs could be observed directly by their reflection and green emission properties. The nucleus and the cytoplasmatic membrane were stained with DAPI and Cell Mask, respectively. Figure 6 shows the results of *in vivo* imaging of treated cells with AgNPs stabilized with compound **3**. A merge image of all the individual channels and a three dimensional reconstruction shows the presence of individual or agglomerated nanoparticles inside the cells (indicated with white arrows). Similar results were obtained with AgNPs stabilized with compound **2**. Due to the cytotoxicity observed in AgNPs stabilized with compound **1**, it was very difficult to visualize nanoparticles in HepG2 cells when cells were treated for 24 h at 100 μg/ml of nanoparticle concentration.

As reported, AgNPs can cross the cell membrane via free shuttle or by damaging the membrane integrity through bonding with thiol-containing proteins (You et al., 2012). We demonstrate here that AgNPs stabilized with compounds **2** and **3** have the ability to penetrate HepG2 cells, while maintaining their fluorescent properties.

#### Silver nanoparticle uptake by flow cytometry

To quantify the cellular uptake of AgNPs, flow cytometry analysis of HepG2 cells was performed. Figure SI4 represents the flow cytometry analysis (shown as histograms of the FL1-X-Chanel or auto-fluorescence of the nanoparticles) of control cells and HepG2 cells incubated with 25 and 100 μg/ml of each AgNP (stabilized with compounds **1**, **2**, and **3**) for 24 h. Results showed an enhancement of cell fluorescence, with a dose-dependent behavior, for the three AgNPs assayed.

## Conclusions

Using photosynthesized emissive AgNPs containing as additives the organic dyes 5-aminoisoquinoline (**1**), 7-amino-4-methylcoumarin (**2**), and 2-aminoanthracene (**3**), *in vivo* and *in vitro* toxicological studies were successfully performed. Regarding *in vivo* studies of oxidative stress, AgNPs with **3** caused larger changes in livers than in intestines with GST level measurements, suggesting the importance of the nanoparticle size in terms of its toxicity. In *in vitro* studies in HepG2 cancer cells, AgNPs **3** displayed less toxicity by cell viability measurements, whereas the AgNps with **1** were shown to be the most toxic. The higher oxidative stress revealed in AgNPs with **3** in *in vivo* studies by GST level, could be related to the nanoparticles and the inner-dye toxicity used as additive. These results highlight the importance of the molecules in the surrounding nanoparticles.

To date results obtained by various research groups have been very variable and depend on the type and nature of AgNPs, the concentration tested, size, route of exposure together with other factors (e.g., aggregation, dissolution) that may influence NP toxicity and difficult inter-study comparisons. For instance, there is the possibility that NP functionalization and mode of delivery influence the presence or absence of Ag (I) ions, which are known to be more toxic than AgNPs themselves.

We found that AgNPs synthesized with additives **1**, **2**, and **3** were able to penetrate tissues and fish cells, as well as HepG2 cells, and still maintained their own fluorescent properties. These results support the idea that the use of emissive AgNPS for biosciences could be an important nano-tool but safety controls need to be ensured.

## Author contributions

All authors conceived and design the experiments, analyzed the data, contributed to discussion and co-wrote the manuscript. Elisabete Oliveira performed the synthesis of silver nanoparticles and characterization. Benito Rodríguez-González performed the TEM and HRTEM measurements. Mário Diniz performed the *in vivo* studies, and Javier Garcia-Pardo and Julia Lorenzo the *in vitro* studies. Carlos Lodeiro and José L. Capelo coordinated and finance the project.

## Conflict of interest statement

The authors declare that the research was conducted in the absence of any commercial or financial relationships that could be construed as a potential conflict of interest.

## References

[B1] Aebi H. (1984). Catalase *in vitro*. Meth. Enzymol. 105, 121–126 10.1016/S0076-6879(84)05016-3 6727660

[B2] Alivisatos A. P. (1996). Semiconductor clusters, nanocrystals, and quantum dots. Science 271, 933–937 10.1126/science.271.5251.933

[B3] Belser K. Slenters T. V. Pfumbidzai C. Upert G. Mirolo L. Fromm K. M. (2009). Silver nanoparticle formation in different sizes induced by peptides identified within split-and-mix libraries. Agew. Chem. Int. Ed. 48, 3661–3664 10.1002/anie.200806265 19373811

[B4] Botsoglou N. A. Fletouris D. J. Papageorgiou G. E. Vassilopoulos V. N. Mantis A. J. Trakatellis A. G. (1994). A rapid, sensitive, and specific thiobarbituric acid method for measuring lipid peroxidation in animal tissues, food, and feedstuff samples. J. Agri. Food Chem. 42, 1931–1937 10.1021/jf00045a019

[B5] Bradford M. M. (1976). A rapid and sensitive method for the quantification of microgram quantities of protein utilizing the principle of protein-dye binding. Anal. Biochem. 72, 248–254 10.1016/0003-2697(76)90527-3 942051

[B6] Chae Y. J. Pham C. H. Lee J. Bae E. Yi J. Gu M. B. (2009). Evaluation of the toxic impact of silver nanoparticles on Japanese medaka (*Oryzias latipes*). Aquat. Toxicol. 94, 320–327 10.1016/j.aquatox.2009.07.019 19699002

[B7] Chau J. L. H. Hsu M. K. Hsieh C. C. Kao C. C. (2005). Microwave plasma synthesis of silver nanopowders. Mater. Lett. 59, 905–908 10.1016/j.matlet.2004.10.068

[B8] Choi J. E. Kim S. Ahn J. H. Youn P. Kang J. S. Park K. (2010). Induction of oxidative stress and apoptosis by silver nanoparticles in the liver of adult zebrafish. Aquat. Toxicol. 100, 151–159 10.1016/j.aquatox.2009.12.012 20060176

[B9] Chow M. K. Zukoski C. F. (1994). Gold sol formation mechanisms: role of colloidal stability. J. Colloid Interface Sci. 165, 97–109 10.1006/jcis.1994.1210

[B10] Correia A. D. Costa M. H. Luis O. J. Livingstone D. R. (2003). Livingstone, Age-related changes in antioxidant enyzme activities, fatty acid composition, and lipid peroxidation in whole body *Gammarus locusta* (Crustacea: Amphipoda). J. Exp. Mar. Biol. Ecol. 289, 83–101 10.1016/S0022-0981(03)00040-6

[B11] Cory A. H. Owen T. C. Barltrop J. A. Cory J. G. (1991). Use of an aqueous soluble tetrazolium/formazan assay for cell growth assays in culture. Cancer Commun. 3, 207–212 1867954 10.3727/095535491820873191

[B12] Dahl J. A. Maddux B. L. S. Hutchinson J. E. (2007). Toward greener nanosynthesis. Chem. Rev. 107, 2228–2269 10.1021/cr050943k 17564480

[B13] Díez I. Pusa M. Kulmala S. Jiang H. Walther A. Goldmann A. S. (2009). Color tunability and electrochemiluminescence of silver nanoclusters. Angew. Chem. 121, 2156–2159 10.1002/ange.200806210 19206134

[B14] Drake P. L. Hazelwood K. (2005). Exposure-related health effects of silver and silver compounds: a review. J. Ann. Occup. Hyg. 49, 575–585 10.1093/annhyg/mei019 15964881

[B15] Evanoff D. D. Jr. Chumanov G. (2005). Synthesis and optical properties of silver nanoparticles and arrays. Chemphyschem. 6, 1221–1231 10.1002/cphc.200500113 15942971

[B16] Farmen E. Mikkelsen H. N. Evensen O. Einset J. Heier L. S. Rosseland B. O. (2012). Acute and sub-lethal effects in juvenile Atlantic salmon exposed to low μ g/L concentrations of Ag nanoparticles. Aquat. Toxicol. 108, 78–84 10.1016/j.aquatox.2011.07.007 22265610

[B17] Faunce T. Watal A. (2010). Nanosilver and global public health: international regulatory issues. Nanomedicine 5, 617–632 10.2217/nnm.10.33 20528456

[B18] Greenberg M. E. Li X.-M. Gugiu B. G. Gu X. Qin J. Salomon R. G. (2008). The lipid Whisker model of the structure of oxidized cell membranes. J. Biol. Chem. 283, 2385–2396 10.1074/jbc.M707348200 18045864

[B19] Habig W. H. Pabst M. J. Jakoby W. B. (1974). Glutathione S-transferases, the first enzymatic step in mercapturic acid formation. J. Biol. Chem. 249, 7130–7139 4436300

[B20] Hayes J. D. Flanagan J. U. Jowsey I. R. (2005). Glutathione transferases. Annu. Rev. Pharmacol. Toxicol. 45, 51–88 10.1146/annurev.pharmtox.45.120403.095857 15822171

[B21] Hussain S. M. Hess K. L. Gearhart J. M. Geiss K. T. Schlager J. J. (2005). *In vitro* toxicity of nanoparticles in BRL 3A rat liver cells. Toxicol. In Vitro 19, 975–983 10.1016/j.tiv.2005.06.034 16125895

[B22] Kiernan J. A. (2008). Histological and Histochemical Methods. Theory and Practice. 4th Edn Bloxham, Scion Publishing Ltd

[B23] Kwok K. W. H. Auffan M. Badireddy A. R. Nelson C. M. Wiesner M. R. Chilkoti A. (2012). Uptake of silver nanoparticles and toxicity to early life stages of Japanese medaka (*Oryzias latipes*): effect of coating materials. Aquat. Toxicol. 120–121, 59–66 10.1016/j.aquatox.2012.04.012 22634717

[B24] Landsdown A. B. G. (2007). Critical observations on the neurotoxicity of silver. Crit. Rev. Toxicol. 37, 237–250 10.1080/10408440601177665 17453933

[B25] Lee P. C. Meisel D. (1982). Adsorption and surface-enhanced raman of dyes on silver and gold sols. J. Phys. Chem. 86, 3391–3395 10.1021/j100214a025

[B26] Li T. Albee B. Alemayehu M. Diaz R. Ingham L. Kamal S. (2010). Comparative toxicity study of Ag, Au, and Ag-Au bimetallic nanoparticles on Daphnia magna. Anal. Bioanal. Chem. 398, 689–700 10.1007/s00216-010-3915-1 20577719

[B27] Liang H. Tang Q. Yu K. Li S. Ke J. (2007). Preparation of metallic silver from Ag2S slurry by direct hydrogen reduction under hydrothermal conditions. Mater. Lett. 61, 1020–1022 10.1016/j.matlet.2006.06.060

[B28] Liz-Marzán L. M. (2006). Tailoring surface plasmons through the morphology and assembly of metal nanoparticles. Langmuir 22, 32–41 10.1021/la0513353 16378396

[B29] Lodeiro C. Capelo J. L. Mejuto J. C. Oliveira E. Santos H. M. Pedras B. (2010). Light and color as analytical detection tools: a journey into the periodic table using polyamines to bio-inspired systems as chemosensors. Chem. Soc. Rev. 39, 2948–2976 10.1039/b819787n 20548989

[B30] Luoma S. (2008). Silver Nanotechnologies and the Environment: Old Problems or New Challenges. Washington, DC: Woodrow Wilson International Center for Scholars Project on Emerging Nanotechnologies or The PEW Charitable Trusts

[B31] Maehly A. C. Chance B. (1954). The assay of catalases and peroxidases. Methods Biochem. Anal. 1, 357–424 10.1002/9780470110171.ch14 13193536

[B32] Maretti L. Billone P. S. Liu Y. Scaiano J. C. (2009). Facile photochemical synthesis and characterization of highly fluorescent silver nanoparticles. J. Am. Chem. Soc. 131, 13972–13980 10.1021/ja900201k 19788331

[B33] Martoja R. Martoja M. (1967). Initiation aux tecniques de l'histologie animal Paris. Masson Cie 1, 345

[B34] Oliveira E. Genovese D. Juris R. Zaccheroni N. Capelo J. L. Raposo M. M. M. (2011a). Bioinspired systems for metal-ion sensing: new emissive peptide probes based on benzo[d]oxazole derivatives and their gold and silica nanoparticles. Inorg. Chem. 50, 8834–8849 10.1021/ic200792t 21848259

[B35] Oliveira E. Nuñez C. Rodríguez-González B. Capelo J. L. Lodeiro C. (2011b). Novel small stable gold nanoparticles bearing fluorescent cysteine-coumarin probes as new metal-modulated chemosensors. Inorg. Chem. 50, 8797–8807 10.1021/ic200664z 21848292

[B36] Pastoriza-Santos I. Liz-Marzán L. M. (2008). Colloidal silver nanoplates. State of the art and future challenges. J. Mater. Chem. 74, 1724–1737 10.1039/b716538b

[B37] Pompa P. P. Martiradonna L. Della Torre A. Della Sala F. Manna L. De Vittorio M. (2006). Metal-enhanced fluorescence of colloidal nanocrystals with nanoscale control. Nat. Nanotechnol. 1, 126–130 10.1038/nnano.2006.93 18654164

[B38] Powers C. M. Slotkin T. A. Seidler F. J. Badireddy A. R. Padilla S. (2011). Silver nanoparticles alter zebrafish development and larval behavior: distinct roles for particle size, coating and composition. Neurotoxicol. Teratol. 33, 708–714 10.1016/j.ntt.2011.02.002 21315816 PMC3112298

[B39] Rao T. U. B. Pradeep T. (2010). Luminescent Ag7 and Ag8 clusters by interfacial synthesis. Angew. Chem. 122, 4017–4021 10.1002/ange.200907120 20408149

[B40] Rodríguez-González B. Mulvaney P. Liz-Marzán L. M. (2007). An electrochemical model for gold colloid formation via citrate reduction. Z. Phys. Chem. 221, 415–426 10.1524/zpch.2007.221.3.415

[B41] Saìnchez-Iglesias A. Pastoriza-Santos I. Peìrez-Juste J. Rodriìguez-Gonzaìlez B. Garciìa De Abajo F. Liz-Marzaìn L. (2006). Synthesis and optical properties of gold nanodecahedra with size control. Adv. Mater. 18, 2529–2534 10.1002/adma.200600475

[B42] Scown T. M. Santos E. M. Johnston B. D. Gaiser B. Baalousha M. Mitov S. (2010). Effects of aqueous exposure to silver nanoparticles of different sizes in rainbow trout. Toxicol. Sci. 115, 521–534 10.1093/toxsci/kfq076 20219766

[B43] Si S. Dinda E. Mandal T. K. (2007). *In Situ* synthesis of gold and silver nanoparticles by using redox-active amphiphiles and their phase transfer to organic solvents. Chem. Eur. J. 13, 9850–9861 10.1002/chem.200701014 17960550

[B44] Sutherlandm W. J. Clout M. Coté I. M. Daszak P. Depledge M. H. Fellman L. (2010). A horizon scan of global conservation issues for 2010. Trends Ecol. Evol. 25, 1–7 10.1016/j.tree.2009.10.003 19939492

[B45] Tam F. Goodrich G. P. Johnson B. R. Halas N. J. (2007). Plasmonic enhancement of molecular fluorescence. Nano. Lett. 7, 496–501 10.1021/nl062901x 17256995

[B46] Vosch T. Antoku Y. Hsiang J. C. Richards C. I. Gonzalez J. I. Dickson R. M. (2007). Strongly emissive individual DNA-encapsulated Ag nanoclusters as single-molecule fluorophores. Proc. Natl. Acad. Sci. U.S.A. 104, 12616–12621 10.1073/pnas.0610677104 17519337 PMC1937515

[B47] Wong H. L. Rauth A. M. Bendayan R. Manias J. L. Ramaswamy M. Liu Z. (2006). A new polymer-lipid hybrid nanoparticle system increases cytotoxicity of doxorubicin against multidrug-resistant human breast cancer cells. Pharm. Res. 23, 1574–1585 10.1007/s11095-006-0282-x 16786442

[B48] You C. Han C. Wang X. Zheng Y. Li Q. Hu X. (2012). The progress of silver nanoparticles in the antibacterial mechanism, clinical application and cytotoxicity. Mol. Biol. Rep. 39, 9193–9201 10.1007/s11033-012-1792-8 22722996 PMC7089021

[B49] Zhang J. Xu S. Kumacheva E. (2005). Photogeneration of fluorescent silver nanoclusters in polymer microgels. Adv. Mater. 17, 2336–2340 10.1002/adma.200501062

