# Supplementary material for: Synthesis of functionalized fluorescent silver nanoparticles and their toxicological effect in aquatic environments (Goldfish) and HEPG2 cells
Source: Front Chem. 2013 Dec 5;1:29. doi: 10.3389/fchem.2013.00029 (PMC3988373; doi:10.3389/fchem.2013.00029)
Supplement: Supplementary file 1 [file Data_Sheet_1.DOC]

Supporting Information

Synthesis of Functionalized Fluorescent Silver Nanoparticles and their toxicological effect in aquatic environments (Goldfish) and HEPG2 cells.

**Elisabete Oliveira*^1^*^,2^*, Hugo M. Santos^1^, Mário Diniz^1^, Javier Garcia-Pardo^3^ ,Julia Lorenzo^3^, Benito Rodríguez-González^4^, José Luis Capelo^1^, Carlos Lodeiro^1^***

^1^BIOSCOPE Group, REQUIMTE, Chemistry Department, FCT-UNL, Monte de Caparica, Portugal.

^2^ Veterinary Science Department, (CECAV), University of Trás-os-Montes and Alto Douro, Vila Real, Portugal.

^3^ Institut de Biotecnologia i Biomedicina and Departament de Bioquímica i de Biologia Molecular, Universitat Autònoma de Barcelona, Bellaterra, Barcelona, Spain.

^4^ C.A.C.T.I, University of Vigo, Campus Universitario, Vigo, Spain.

***Correspondence:** Elisabete Oliveira, Chemistry Department, REQUIMTE, Faculty of science and Technology, University NOVA of Lisbon, CAPARICA Campus, 2825-366 Portugal

email: ej.oliveira@fct.unl.pt

Carlos Lodeiro, Chemistry Department, REQUIMTE, Faculty of Science and Technology, University NOVA of Lisbon, CAPARICA Campus, 2825-366 Portugal

email: cle@fct.unl.pt.

**Figures**

**Figure SI1** - (A) Absorption and (B) emission spectra of the AgNPs formation with time containing as stabilizer compound **2**, in a THF solution, T=298 K, λ_exc_ = 430 nm.

**Figure SI2**. Intraperitoneal injection of 0.15 mg/mL of compounds **1**, **2**, or **3** (1mL THF diluted to 10 mL PBS). (**I**) GST, (**II**) CAT and (**III**) MDA concentrations in the liver and intestine tissues of *Carassius auratus* treated with AgNPs for 48 h. Data are presented as means±s.d. No statistic significant differences were observed (p < 0.05) in comparison to controls. Control: PBS (phosphate buffer solution), n=3.

***
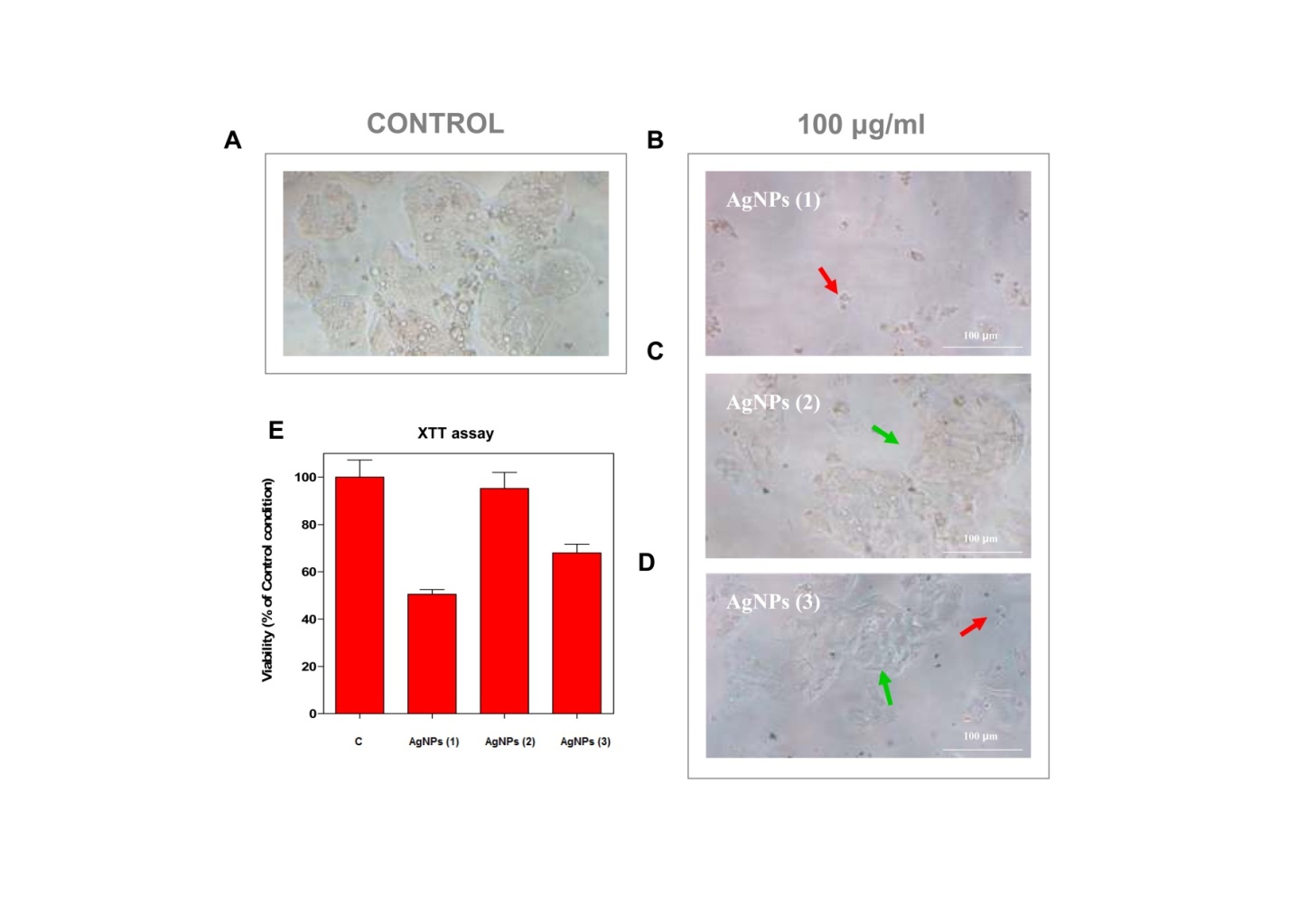
***

**Figure SI3 -**  Comparative cytotoxicity against HepG2 cells after 72 h of treatment with AgNPs (stabilized with compounds **1**, **2** and **3**) at 100 μg/ml. Representative optical microscopy images of control cells (A) and AgNPs treated cells (B, C, D) are shown. Green and red arrows indicate viable and non-viable HepG2 cells respectively. Cell viability of HepG2 cells was evaluated by XTT assay (E).


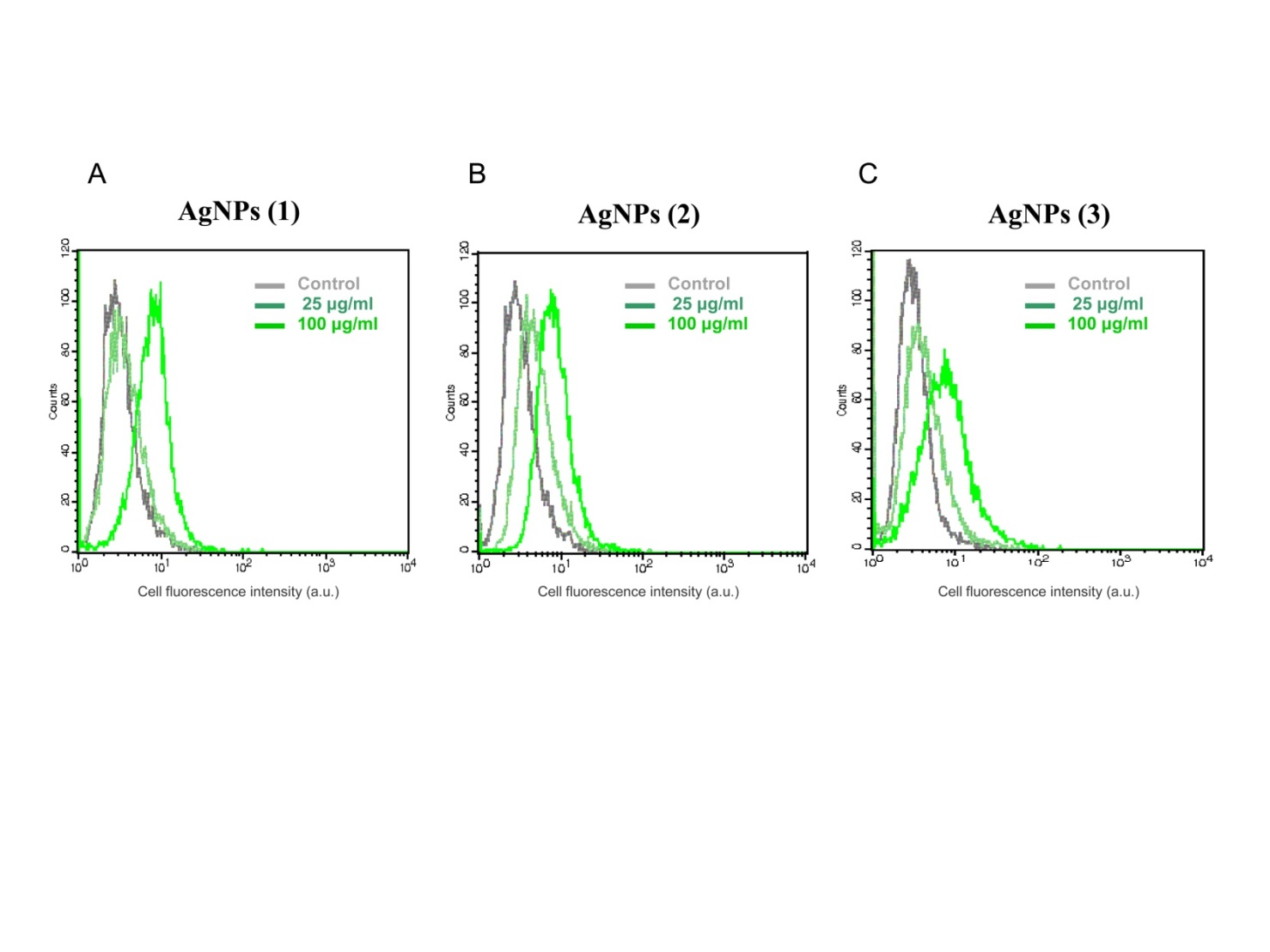


**Figure SI4 -**  Flow cytometric analyses of HEK293-T cells exposed to 25 and 100 μg/ml of AgNPs stabilized with compound **1** (A), compound **2** (B) and compound **3** (C).
